# Food insecurity among Romani adults with chronic illness in Jordan: prevalence, predictors, and public health implications

**DOI:** 10.3389/fpubh.2025.1639325

**Published:** 2025-10-07

**Authors:** Mohammad Othman Abudari, Nadin Abdel Razeq, Mahmoud Al-Hussami, Abdullah Algunmeeyn, Manar Abu-Abbas

**Affiliations:** ^1^School of Nursing, The University of Jordan, Amman, Jordan; ^2^School of Nursing, Yarmouk University, Irbid, Jordan

**Keywords:** Romani population, food insecurity, chronic disease, vulnerable populations, Jordan

## Abstract

**Background:**

The Roma community in Jordan, as in other parts of the world, often resides in informal settlements with limited access to employment, education, and basic services, increasing their vulnerability to food insecurity and poor health.

**Objective:**

This study examined the prevalence and predictors of food insecurity among Roma adults in Jordan living with chronic diseases.

**Methods:**

A cross-sectional descriptive study was conducted among 347 Roma individuals with at least one chronic illness across four governorates. Data on demographics, health behaviors, and food security status were collected through structured questionnaire interviews. Analysis was performed using descriptive statistics and binary logistic regression to identify associated factors.

**Results:**

Food insecurity was widespread, with 75.6% of participants reporting low food security and 14.9% very low food security. Logistic regression identified key predictors: having two or more chronic conditions increased risk (OR = 2.21, 95% CI [1.13, 4.32], *p* = 0.021), as did being divorced/widowed (OR = 6.56, 95% CI [1.05, 41.05], *p* = 0.044). Residence in Amman (OR = 0.04, 95% CI [0.01, 0.17], *p* < 0.001) and Madaba (OR = 0.09, 95% CI [0.02, 0.44], *p* = 0.003) reduced risk. Difficult healthcare access (OR = 3.50, 95% CI [1.29, 9.46], *p* = 0.014) elevated risk, while good/excellent self-rated health was protective (OR = 0.22, 95% CI [0.09, 0.52], *p* = 0.001).

**Conclusion:**

Food insecurity is highly prevalent among Roma with chronic diseases in Jordan. Addressing this issue through targeted nutritional and healthcare interventions is vital to reduce disease burden and health disparities in this marginalized population.

## 1 Introduction

Food insecurity—defined as limited or uncertain access to nutritionally adequate and safe foods—is a critical global health challenge, affecting an estimated 2.3 billion people worldwide in 2024 (1, 2). The prevalence of food insecurity remains disproportionately high in low- and middle-income countries, driven by conflict and insecurity, weather extremes, and economic shocks (3).

It disproportionately impacts marginalized and socioeconomically disadvantaged populations, often reinforcing cycles of poverty, poor health, and social exclusion (4).

Ethnic minority groups such as the Romani population face heightened vulnerability to food insecurity due to entrenched structural inequalities. The Romani, often referred to as “an-Nuar” in Jordan, represent one of the most marginalized ethnic minorities globally, with significant social, economic, and health disparities documented across Europe and the Middle East (5, 6).

Economic hardships force many Romani to rely on informal activities such as collecting scrap, street vending, or seasonal agricultural work, as formal employment opportunities remain inaccessible due to discrimination, low education levels, and social exclusion (7, 8). Educational exclusion is prevalent, with many Romani children not attending school, resulting in alarmingly high illiteracy rates (7). These conditions significantly compromise their food security, particularly among those managing chronic illnesses that require consistent nutritional and medical care (7, 9).

Food insecurity has been extensively linked to adverse health outcomes, including obesity, type 2 diabetes, hypertension, cardiovascular diseases, and poor mental health (10, 11). Among individuals with chronic conditions, food insecurity can exacerbate disease progression by limiting access to appropriate diets, medications, and routine health services. This lack of preventive care may precipitate adverse health events, such as disease complications, that require urgent care, thereby increasing the likelihood of using health services for adverse health events (12).

While the health implications of food insecurity have been widely studied in general and low-income populations, little is known about these dynamics in ethnically marginalized groups, particularly in Middle Eastern contexts. While these dynamics are globally documented, no empirical studies examine food insecurity among chronically ill Romani in the Middle East—a critical gap given Jordan’s unique socioeconomic context and the community’s compounded vulnerability.

The Romani communities in Europe and Jordan and the broader Middle East trace their origins to northwest India, as evidenced by linguistic and genetic studies indicating a westward migration between the 5th and 11th centuries (13, 14). While the Romani in Europe now number approximately 10–12 million across Europe, the Romani in Jordan remain smaller and more dispersed, with the Jordanian population estimated at around 70,000 (7, 15). Despite this shared ancestry, the two communities differ significantly in terms of language retention, cultural visibility, and policy inclusion. European Romani have preserved the Romani language and developed a transnational identity supported by legal recognition and advocacy platforms at both national and EU levels (16). In contrast, Romani language use in the Middle East has largely been supplanted by Arabic, and Romani communities face deep-rooted stigmatization, often concealing their identity to avoid discrimination (17). Socioeconomic hardship persists in both contexts—approximately 80% of European Roma live below the poverty line and face systemic exclusion in education, housing, and employment (6); similarly, Romani in Jordan and neighboring countries endure poverty and informal employment (7).

The Romani population in Jordan represents a unique intersection of ethnic, economic, and health vulnerabilities. Despite their long-standing presence in the country, they remain largely invisible in national data systems and underserved by mainstream public health initiatives (9). Importantly, no empirical studies to date have examined food insecurity among Romani adults in Jordan, particularly those living with chronic illnesses—a gap that hinders the development of targeted and inclusive health policies.

To address this critical knowledge gap, the present study aimed to:

1. Estimate the prevalence of food insecurity among chronically ill Romani adults in Jordan;
2. Identify key socio-demographic, health-related, and geographic predictors of food insecurity; and

Focusing on chronically ill Romani is imperative: a synergy chronic diseases amplify financial strain and nutritional needs, yet intersect with ethnic marginalization; unexplored in Jordan.

By focusing on a highly underserved population, this study contributes to the global discourse on health equity and provides evidence to inform culturally responsive interventions designed to improve the wellbeing of ethnically marginalized groups in LMIC settings.

## 2 Materials and methods

### 2.1 Study design and setting

This study used a descriptive, cross-sectional design to explore the prevalence and predictors of food insecurity among chronically ill Romani adults in Jordan. Data were collected from four governorates—Amman, Irbid, Madaba, and Al-Mafraq—chosen because they host sizable Romani populations and capture different urban–rural contexts. Amman, the capital, is a highly urbanized city with advanced infrastructure and wide access to services. Madaba, by contrast, is a smaller urban center, while Irbid, the country’s second-largest city, combines both urban and peri-urban features. Al-Mafraq, in turn, is more remote and underserved compared to other regions, with large rural areas and limited service availability. Together, these regions provide a diverse backdrop of socioeconomic and healthcare conditions, allowing us to examine how geography shapes disparities in food security.

### 2.2 Study population and eligibility criteria

The target population consisted of self-identified Romani adults (≥18 years) residing in the selected regions with a confirmed or self-reported diagnosis of at least one chronic illness lasting 1 year or more, requiring ongoing medical care or limiting daily activities, such as hypertension, type 2 diabetes, cardiovascular diseases, cerebrovascular diseases, chronic respiratory conditions, or neurological disorders. Exclusion criteria included severe cognitive or psychiatric impairments (e.g., schizophrenia, dementia) that could interfere with informed consent or the reliability of survey responses. Exclusions were based on self-reported physician diagnoses and cognitive status assessed by trained researchers using the 6-Item Cognitive Impairment Test (6CIT) during recruitment. This validated brief tool evaluates orientation, attention, and short-term memory, with a maximum score of 28. Participants scoring ≥10 were excluded based on established thresholds indicating moderate-to-severe cognitive impairment (18).

### 2.3 Sampling strategy and sample size

Given the absence of an official Romani registry, convenience sampling was utilized. Community engagement strategies, including collaboration with recognized Romani leaders, facilitated participant recruitment and access to settlements. Each district leader assisted in identifying eligible participants and in assigning interpreters or facilitators as needed. The overrepresentation of Amman-based participants (53.9%) mirrors the spatial distribution of Jordan’s Romani population, who predominantly cluster in the capital’s informal settlements due to socioeconomic push-pull factors (7). While this limits granular geographic comparisons, our analysis accounts for regional variation via stratified models and confirms robustness through sensitivity testing (see Results).

*A priori* sample size estimation was conducted using G*Power 3.1 software. Based on a binary logistic regression model, with an alpha level of 0.05, power of 80%, and an anticipated odds ratio (OR) of 1.5, the minimum required sample size was calculated to be 308 participants. To accommodate potential non-responses or missing data, a 10% buffer was added, resulting in a target of 350 participants. After excluding three incomplete surveys, the final analytical sample comprised 347 individuals.

### 2.4 Data collection procedures

Data were collected between January and February 2021 via structured, face-to-face interviews administered by the principal investigator and a trained nursing graduate student. Interviews were conducted in private settings—typically within participants’ homes or tents—to ensure confidentiality and comfort. A culturally adapted Arabic-language questionnaire was used, developed based on validated tools and pretested among 50 Romani individuals to ensure clarity, reliability, and cultural appropriateness.

Before participation, voluntary verbal informed consent was obtained, in accordance with ethical protocols for populations with high illiteracy. No identifying information was collected. Interviews lasted approximately 15–20 min and were conducted with interpreter support as needed.

### 2.5 Measures and instruments

#### 2.5.1 Food insecurity assessment

Food security status was evaluated using the 10-item U. S. Household Food Security Survey Module (HFSSM), which assesses adult-level food insecurity over the past 12 months (19). This tool captures both qualitative and quantitative aspects of food access, including worry about food sufficiency and actual reduction or skipping of meals due to financial constraints. Total scores were classified as follows:

0: High food security

1–2: Marginal food security.

3–5: Low food security.

6–10: Very low food security.

For logistic regression, the outcome variable was dichotomized as “Low/Very Low Food Security” (scores 3–10) versus “High/Marginal Food Security” (scores 0–2), consistent with USDA analytic standards.

#### 2.5.2 Sociodemographic variables

Data were collected on age, sex, marital status, household size (categorized as <5, 5–8, >8), place of residence (Amman, Irbid, Al-Mafraq, or Madaba), employment status, and educational attainment. Participants’ marital status was grouped into three categories: never married, currently married, and previously married (including divorced or widowed). Education level was categorized as “unable to read and write” or “completed at least secondary education.” In our sample, only one participant was literate. This reflects the extremely low school attendance among Romani children in Jordan, which results in most adults lacking even primary-level literacy skills.

#### 2.5.3 Health-related variables

Participants self-reported their primary chronic illness, age at diagnosis, number of chronic conditions, disease duration (<4 years or ≥4 years), and health insurance coverage (yes/no). Smoking and alcohol use were assessed with categorical frequency items.

#### 2.5.4 Canadian community health survey instruments

Four validated subscales from the Canadian Community Health Survey (CCHS) (20) were employed to evaluate:

1. Perceived Accessibility of Healthcare—measured using the 16-item Barriers to Access Scale (score range 0–64; scores >32 indicated poor access).
2. Self-Rated General and Mental Health—using 4-point Likert scales from “poor” (0) to “excellent” (4), later dichotomized as “poor/fair” vs. “good/excellent.”
3. Health Services Utilization—whether the participant accessed care in the past 12 months (yes/no).
4. Unmet Healthcare Needs—measured using a 10-item scale assessing how often health needs were unmet due to cost, availability, or other factors.

### 2.6 Translation, content validity assessment, and reliability analysis

All instruments were translated and culturally adapted into Arabic following the World Health Organization’s recommended protocol for adapting and translating research instruments (21). The process involved forward translation by bilingual experts, expert panel review, back-translation, and cognitive pretesting with the target population to ensure conceptual and functional equivalence.

#### 2.6.1 Content validity assessment

As part of the cultural adaptation process, all instruments underwent expert review by five bilingual professionals specializing in nursing, public health, and health services research. Experts assessed each item’s clarity and relevance using a 4-point Likert scale (1 = not clear/relevant, 4 = highly clear/relevant).

##### 2.6.1.1 Perceived accessibility of healthcare (16 items)

Item-Level Content Validity Index (I-CVI) values ranged from 0.80 to 1.00, with a Scale-Level Content Validity Index Average (S-CVI/Ave) of 0.93 for both clarity and relevance. Experts suggested minor cultural rewording for one item; clarifying the term “family doctor” as “primary care physician.”

##### 2.6.1.2 Unmet healthcare needs (10 items)

I-CVI values ranged from 0.80 to 1.00, with an S-CVI/Ave of 0.92. Minor adjustments were recommended for phrasing items related to cost and availability to reflect local healthcare system terminology.

##### 2.6.1.3 10-item U.S. household food security survey module (HFSSM)

I-CVI values ranged from 0.80 to 1.00, with an S-CVI/Ave of 0.94 for both clarity and relevance. Minor rewording suggestions were noted for three items without altering their core meaning.

##### 2.6.1.4 Self-rated general and mental health (2 items) and health services utilization (1 item)

For these single-item or binary-response indicators, formal I-CVI calculation was not applicable. However, experts confirmed their linguistic clarity and cultural appropriateness. All items were deemed conceptually equivalent to their original English versions.

The I-CVI and S-CVI/Ave values reported for all multi-item scales meet or exceed internationally accepted benchmarks for health-related research instruments. According to Polit and Beck, I-CVI values ≥ 0.78 and S-CVI/Ave values ≥ 0.80 indicate acceptable content validity (22).

#### 2.6.2 Reliability analysis

Internal consistency reliability was assessed using Cronbach’s alpha based on pilot data collected from 50 Romani adults from the target population. The following reliability coefficients were obtained: Perceived Accessibility of Healthcare: Cronbach’s alpha = 0.72, Unmet Healthcare Needs: Cronbach’s alpha = 0.76, and 10-Item HFSSM: Cronbach’s alpha = 0.78.

All values exceeded the commonly accepted threshold of 0.70 for group-level comparisons in health and social sciences research, as recommended by Taber (23). Examination of alpha-if-item deleted values showed no substantial improvement across these scales (range: 0.75–0.80), supporting the retention of all original items.

For the Self-Rated General and Mental Health and Health Services Utilization items, internal consistency reliability was not calculated because these represent single-item or categorical variables, for which Cronbach’s alpha is not applicable.

Overall, the translation, adaptation, and reliability testing procedures ensured both linguistic and psychometric equivalence of the adapted instruments.

### 2.7 Ethical considerations

The study received ethical clearance from the Research Ethics Committee affiliated with the University of Jordan. The study complied with the principles of the Declaration of Helsinki and national regulations. Given the high illiteracy rate, verbal informed consent was obtained with comprehensive oral explanations of the study’s purpose, risks, and participant rights. Privacy, anonymity, and confidentiality were strictly maintained throughout the study.

### 2.8 Data analysis

All data were processed using IBM SPSS Statistics version 26. Summary statistics—including proportions, averages, and standard deviations—were calculated to describe participant profiles. Relationships between food insecurity and key variables were initially examined through chi-square tests and independent t-tests. Binary logistic regression analysis was conducted to identify independent predictors of food insecurity. Variables with *p* < 0.10 in bivariate analyses were entered into the multivariable model. Odds ratios (ORs) with 95% confidence intervals (CIs) were reported. Multicollinearity was assessed using variance inflation factor (VIF), and model fit was evaluated using the Hosmer–Lemeshow test. A two-tailed *p*-value of <0.05 was considered statistically significant.

## 3 Results

### 3.1 Participant characteristics

A total of 347 Romani adults with chronic illnesses participated in the study. The sample was nearly gender-balanced, with 52.4% men and 47.6% women participants. The mean age was 48.2 years (SD = 13.5), with the majority (59.7%) falling within the 41–60 age group. The majority of individuals reported being currently married (89.1%), with over half residing in the capital city, Amman (53.9%).

Household sizes were generally large, with an average of 6.8 members (SD = 3.4); 30% reported living in households with more than eight individuals. Educational attainment was strikingly low, with 99.7% reporting illiteracy. Unemployment was high, with only 18% reporting current employment (Table 1).

**Table 1:** Characteristics of Romani participants living with chronic illnesses in Jordan (*N* = 347).

Characteristic	Category	Frequency (*n*)	Percentage (%)
Sex	Men	182	52.4
	Women	165	47.6
Age range (Years) (Mean = 48.2, SD = 13.5)	<21 years	14	4.0
	21–40 years	82	23.6
	41–60 years	207	59.7
	>60 years	44	12.7
Marital status	Single	18	5.3
	Currently married	303	89.1
	Separated/Divorced	6	1.8
	Widowed	13	3.8
Region of residence	Al-Mafraq	64	18.4
	Ma’daba	38	11.0
	Irbid	58	16.7
	Amman	187	53.9
Household size (Mean = 6.8, SD = 3.4)	Less than 5 members	84	24.2
	5–8 members	159	45.8
	Over 8 members	104	30.0
Education level	Unable to read or write	346	99.7
	Completed secondary School	1	0.3
Employment	Currently Employed	58	18.0
	Not Employed	265	82.0

### 3.2 Health profile

The most frequently reported *primary* chronic illnesses were type 2 diabetes (38.4%) and elevated blood pressure (36.8%), followed by stroke-related disorders (7.4%) and neurological impairments (6.0%). Smaller proportions reported heart disease (3.9%), lung disease (3.0%), or other chronic conditions (4.6%). On average, participants had been living with their chronic condition for 5.1 years (SD = 4.6), and 21.6% had lived with it for more than 6 years. Over half (55.0%) lacked health insurance coverage. Most participants were non-smokers (64.0%) and reported no alcohol consumption (98.8%). Regarding self-perceived health, 14.1% rated their general health as poor or fair, and 38.3% rated their mental health as poor or fair. In the past 12 months, 75.2% reported utilizing healthcare services, yet 81.6% experienced unmet healthcare needs, often due to cost, availability, or other access barriers. Perceived access to healthcare was generally poor, with 81.0% reporting difficulty obtaining needed services. Most participants (63.5%) were living with two or more chronic conditions, underscoring the complex health needs of this population (Table 2).

**Table 2:** Health practices and key health indicators of Romani participants (*N* = 347).

Health indicators	Category	Frequency (*n*)	Percentage (%)
Chronic disease types^$^	Diabetes	218	38.4
	Elevated Blood Pressure	209	36.8
	Stroke-Related Disorders	42	7.4
	Neurological Impairments	34	6.0
	Heart Diseases	22	3.9
	Lung Diseases	17	3.0
	Other Chronic Conditions	26	4.6
Cigarette use^‡‡^	Non-smoker	222	64.0
	<20 cigarettes per day	90	25.9
	21–40 cigarettes per day	26	7.5
	>40 cigarettes per day	8	2.3
Shisha use	Does not use	337	97.1
	Once per day	6	1.7
	2–6 times weekly	3	0.9
Alcohol consumption	No alcohol consumption	343	98.8
	Once daily	2	0.6
	Once weekly	1	0.3
Chronic disease duration (Mean = 5.1, SD = 4.6)	1–3 years	167	48.1
	4–6 years	105	30.3
	Over 6 years	75	21.6
Health insurance	No Coverage	191	55.0
	Covered	156	45.0
Self-rated general health^*^	Poor/Fair	49	14.1
	Good/Excellent	298	85.9
Self-rated mental health^*^	Poor/Fair	133	38.3
	Good/Excellent	214	61.7
Healthcare utilization^**^	No	86	24.8
	Yes	261	75.2
Number of chronic conditions	One chronic condition	127	36.5
	Two or more chronic conditions	220	63.5
Unmet healthcare needs^†^	Never/Rarely	64	18.4
	Sometimes/Often/Always	283	81.6
Perceived healthcare access^‡^	Poor/Difficult	281	81.0
	Easy	66	19.0

$ It should be noted that these figures represent only the primary chronic condition identified for each participant and therefore do not reflect the prevalence of all chronic conditions present in the sample.

* Self-rated general and mental health — Assessed using the questions: “In general, how would you rate your overall health?” and “In general, how would you rate your mental health?” Responses were recorded on a 4-point Likert scale (0 = poor to 4 = excellent) and subsequently dichotomized into “poor/fair” vs. “good/excellent.”

† Unmet healthcare needs — Assessed using the question: “During the past 12 months, was there ever a time when you felt that you needed healthcare, but did not receive it?” Measured with a 10-item scale (score range 0–40); scores > 20 indicated unmet needs. Response options were dichotomized as “sometimes/often/always” vs. “never/rarely.”

‡ Perceived healthcare access — Assessed using the question: “During the past 12 months, how easy or difficult was it for you to obtain needed healthcare services?” Measured with the 16-item Barriers to Access Scale (score range 0–64); scores > 32 indicated poor access.

** Healthcare utilization — Assessed using the question: “During the past 12 months, have you received any healthcare services?” Responses were coded as yes/no.

‡‡ Smoking status by gender: Of the 124 current smokers, 112 were males (90.3%) and 12 were females (9.7%). Among the 222 non-smokers, 73 were males (32.9%) and 149 were females (67.1%).

### 3.3 Prevalence of food insecurity

**Figure 1:**
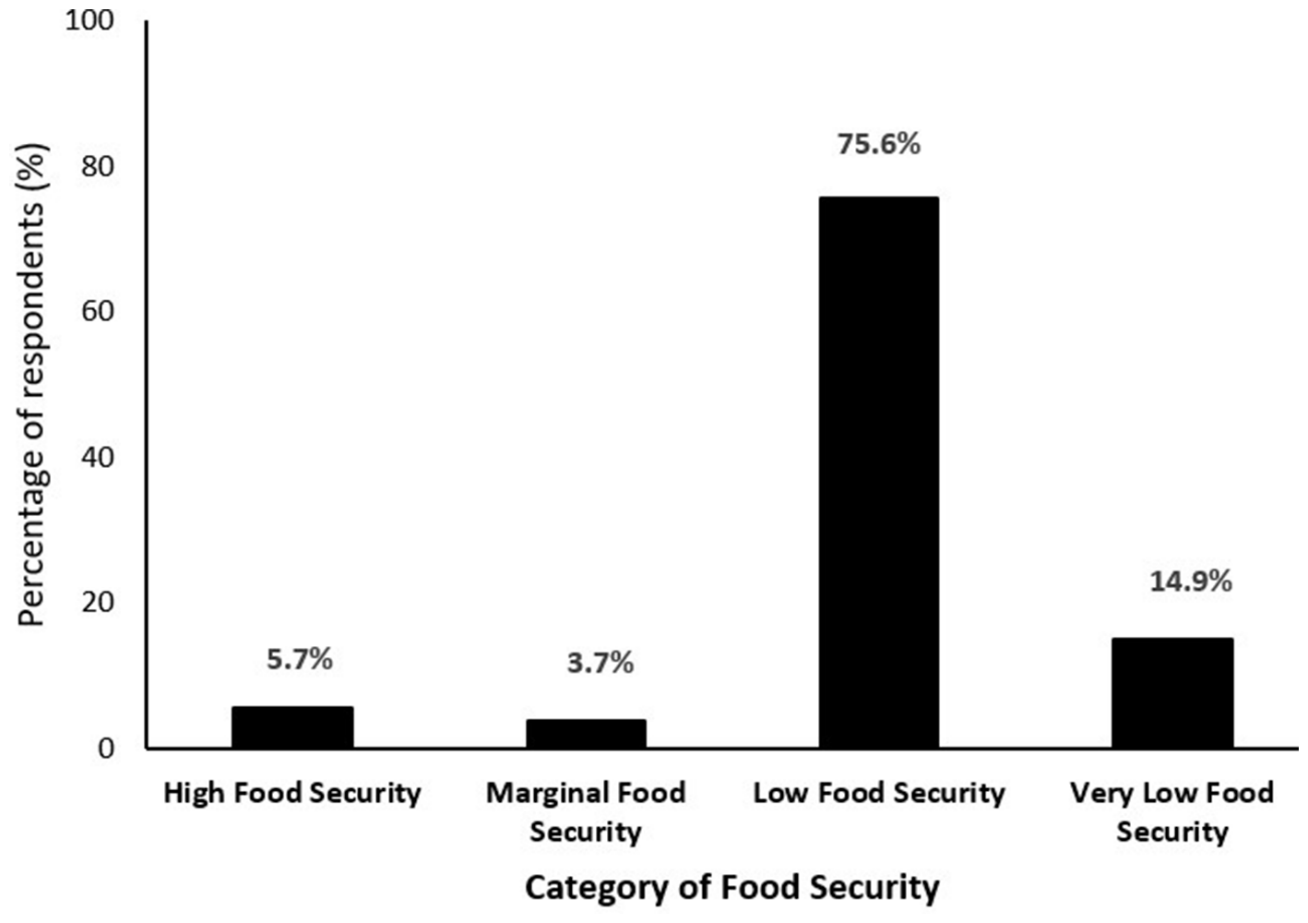
Food security status among Romani population in Jordan with chronic diseases.

As shown in Figure 1, 90.5% of participants experienced some level of food insecurity. Specifically, 75.6% reported low food security, while an additional 14.9% reported very low food security. Only 5.7% were identified as food secure, and 3.7% as marginally food secure. This indicates that nearly all participants lived with inadequate access to nutritious and sufficient food, with a substantial proportion experiencing severe deprivation.

### 3.4 Predictors of food insecurity

Results from the binary logistic regression analysis are presented in Table 3. Several factors emerged as significant predictors of food insecurity.

- Number of chronic conditions: Participants with two or more chronic diseases had more than twice the risk of food insecurity compared to those with only one condition (OR = 2.21, 95% CI: 1.13–4.32, *p* = 0.021).
- Residence: Urban residence was protective. Individuals living in Amman (OR = 0.04, 95% CI: 0.01–0.17, *p* < 0.001) and Madaba (OR = 0.09, 95% CI: 0.02–0.44, *p* = 0.003) had significantly lower odds of food insecurity compared to those in Al-Mafraq. Residence in Irbid did not show a significant association (OR = 0.50, 95% CI: 0.08–3.29, *p* = 0.472).
- Marital status: Divorced or widowed individuals were at markedly increased risk of food insecurity compared to single individuals (OR = 6.56, 95% CI: 1.05–41.05, *p* = 0.044). Married participants also had elevated, but not statistically significant, odds (OR = 2.40, 95% CI: 0.66–8.75, *p* = 0.185).
- Healthcare access: Difficulty accessing healthcare services was strongly connected with food insecurity (OR = 3.50, 95% CI: 1.29–9.46, *p* = 0.014).
- Self-rated general health: Participants who rated their health as good or excellent had significantly lower odds of food insecurity (OR = 0.22, 95% CI: 0.09–0.52, *p* = 0.001), suggesting that better health perception correlates with better food access and stability.

**Table 3:** Logistic regression results for food insecurity with all variables (*N* = 347).

Variable	Category (Reference)	B	SE	Wald	*p*-value	OR	95% CI for OR
Sex	Female (Male ref)	−0.344	0.398	0.747	0.387	1.411	[0.646, 3.079]
Age group	40–59 years (<40 ref)	−0.095	0.434	0.048	0.826	0.909	[0.389, 2.127]
	≥ 60 years (<40 ref)	−0.542	0.570	0.905	0.341	0.581	[0.190, 1.777]
Number of chronic conditions	Two or more (One ref)	0.791	0.343	5.327	0.021	2.205	[1.127, 4.316]
Marital status	Married (Single ref)	0.875	0.660	1.758	0.185	2.400	[0.658, 8.753]
	Divorced/Widowed (Single ref)	1.881	0.936	4.038	0.044	6.557	[1.047, 41.050]
Region of residence	Madaba (Al-Mafraq ref)	−2.459	0.841	8.554	0.003	0.086	[0.016, 0.444]
	Irbid (Al-Mafraq ref)	−0.690	0.960	0.517	0.472	0.501	[0.076, 3.294]
	Amman (Al-Mafraq ref)	−3.265	0.762	18.383	0.000	0.038	[0.009, 0.170]
Employment status	Working (Not working ref)	−0.354	0.433	0.670	0.413	0.702	[0.300, 1.639]
Chronic disease duration	≥ 4 years (<4 years ref)	0.439	0.324	1.841	0.175	1.552	[0.823, 2.926]
Healthcare access	Difficult (Easy ref)	1.252	0.508	6.071	0.014	3.496	[1.292, 9.464]
Self-rated general health	Good/Excellent (Poor/Fair ref)	−1.517	0.444	11.658	0.001	0.219	[0.092, 0.524]
Self-rated mental health	Good/Excellent (Poor/Fair ref)	−0.397	0.313	1.608	0.205	0.672	[0.364, 1.242]
Household size	> 5 members (≤ 5 ref)	−0.097	0.406	0.057	0.811	0.908	[0.410, 2.011]
Healthcare utilization	Yes (No ref)	−0.357	0.316	1.276	0.259	0.700	[0.376, 1.300]
Unmet healthcare needs	Sometimes/Most/Always (Never/Rarely ref)	0.441	0.505	0.761	0.383	1.554	[0.577, 4.185]
Health insurance	Covered (No insurance ref)	0.195	0.330	0.350	0.554	1.215	[0.637, 2.319]
Cigarette smoking status	Smoker (non-smoker ref)	0.089	0.367	0.059	0.808	1.093	[0.533, 2.243]

B, unstandardized regression coefficient; SE, standard error; Wald, Wald chi-square test statistic; OR, odds ratio; CI, confidence interval (95%). Statistical significance was set at *p* < 0.05. Reference categories: Male (Sex), <40 years (Age), One chronic condition (Chronic Conditions), Single (Marital Status), Al-Mafraq (Region), Not working (Employment), <4 years (Chronic Disease Duration), Easy (Healthcare Access), Poor/Fair (Self-rated Health), ≤5 members (Household Size), No (Healthcare Utilization), Never/Rarely (Unmet Healthcare Needs), and No Insurance (Health Insurance).

Other variables — including sex, age, employment status, mental health, health insurance, healthcare utilization, household size, smoking status chronic disease duration and unmet healthcare needs — were not statistically significantly associated with food insecurity in the bivariate logistic regression analyses. The overall logistic model was statistically significant overall [χ^2^ (19, *N* = 341) = 136.459, *p* < 0.001] and explained 46.1% of the variance in food insecurity status (Nagelkerke R^2^). The model correctly classified 79.1% of cases, indicating strong predictive accuracy.

## 4 Discussion

This study provides the first empirical evidence on the prevalence and predictors of food insecurity among chronically ill Romani adults in Jordan—a population facing multiple, intersecting forms of marginalization. The findings reveal an alarmingly high burden of food insecurity, with over 90% of participants reporting either low or very low food security. Several socio-demographic and health-related factors were significantly associated with increased risk, including longer disease duration, multiple chronic conditions, poor self-rated health, rural residence, and difficulty accessing healthcare.

### 4.1 Social and structural determinants of food insecurity

This study provides compelling evidence of the disproportionate burden of food insecurity among chronically ill Romani adults in Jordan, revealing a crisis that far exceeds national norms. While food insecurity in the general Jordanian population has been estimated between 13.9 and 23.1% during the COVID-19 pandemic (24, 25), the rate among our study participants (75.6% low food security and 14.9% very low) represents a stark disparity that demands urgent public health attention.

These findings are consistent with global literature documenting the heightened vulnerability of marginalized ethnic communities, including Roma populations, to food insecurity (5, 26, 27). These findings align with studies from across Europe, which reveal that Roma communities often face much higher levels of food insecurity and poorer diets. This stems from a mix of economic hardship and systemic barriers, as seen in countries like Spain, Romania, and Hungary, as well as in broader EU research (27–31).

### 4.2 Chronic illness and food insecurity: a bi-directional relationship

These findings are consistent with the well-documented bi-directional relationship between chronic illness and food insecurity, whereby poor dietary intake and instability in food access can exacerbate disease progression, while illness itself can diminish income-generating capacity and strain household resources (10, 32, 33).

### 4.3 Geographic and social inequities

Geographic disparities also emerged as a key social determinant. Participants living in Amman and Madaba (urban areas with relatively better infrastructure and access to services) had significantly lower odds of food insecurity compared to those in Al-Mafraq, a more remote and underserved region. These findings mirror patterns observed in other LMICs, where rural and peri-urban residents face heightened vulnerability due to geographic isolation, fragile employment markets, and weak social protection networks (34).

Marital status further influenced outcomes. Divorced or widowed participants were more likely to experience food insecurity, likely due to diminished household income, caregiving burdens, and loss of social support. This trend aligns with evidence that female-headed and single-parent households are particularly susceptible to food deprivation across both high-income and low-income settings (35, 36).

### 4.4 Intersecting structural barriers and cultural context

It is important to view these findings through the lens of intersectional structural disadvantage. The Romani community in Jordan faces multi-layered barriers, including legal invisibility, discrimination, informal employment, and stigma factors that collectively undermine household stability and food security. Although gender, age, and household size did not show significant associations in our model, this may reflect sample homogeneity and unmeasured protective social dynamics, such as intergenerational caregiving and food-sharing practices within extended families.

Further, although detailed income data could not be reliably collected due to the informal and undocumented nature of economic activity in Romani communities, proxy indicators such as employment status and education provide insight into economic vulnerability. Nonetheless, we acknowledge this as a limitation and highlight the need for culturally sensitive tools to better capture economic precarity in marginalized populations.

Together, these results suggest that improving food security for Romani populations with chronic illness will require multi-sectoral approaches that address both immediate nutritional needs and the broader social, health, and economic exclusion they face.

### 4.5 Education and employment

Illiteracy was prevalent among participants 99.7%, far exceeding Jordan’s national illiteracy rate of 4.5% (37). Illiteracy restricts access to healthcare information and effective chronic disease management, compounding food insecurity. These findings align with global research showing educational barriers among Roma communities due to poverty and systemic exclusion (7, 38).

Employment challenges were equally pronounced, with 82% of working-age participants unemployed compared to Jordan’s national unemployment rate of 21.4% (39). Roma employment is often informal and precarious, including activities like scrap collection and street vending. Logistic regression revealed no significant correlation between employment and food security (*p* = 0.413) which may reflect not only the unstable nature of Roma employment but also the buffering effect of extended kinship structures, where shared caregiving, pooled informal income, and collective resource management help mitigate food insecurity risks despite individual unemployment (40). Addressing these disparities requires vocational training, literacy programs, and anti-discrimination policies to create pathways to stable employment.

### 4.6 Healthcare access and food security

Difficulty in accessing healthcare services was another key predictor. Participants reporting poor access to outpatient care had significantly higher odds of food insecurity, reinforcing the interconnectedness of healthcare and nutrition. Healthcare barriers—including lack of insurance, transportation challenges, and cost—may divert resources away from food expenditures or delay disease management, exacerbating both malnutrition and morbidity (41). Integrated health and nutrition support services, particularly in underserved communities, are critical for breaking this cycle.

### 4.7 Implications for policy and practice

The findings of this study have direct implications for public health planning in Jordan and similar LMICs. First, routine screening for food insecurity should be integrated into chronic disease management, particularly in clinics serving vulnerable populations. Second, culturally tailored interventions—such as mobile food pantries, targeted cash transfers, or community kitchens—should be implemented in Romani settlements, taking into account linguistic, cultural, and geographic barriers. Third, broader structural reforms are necessary to tackle the underlying causes of food insecurity among ethnic minorities. These include expanding access to universal health coverage, improving infrastructure in informal settlements, and providing vocational training programs aimed at improving employment opportunities. Intersectoral collaboration—linking health, nutrition, education, and social protection services—is essential for achieving sustainable progress.

### 4.8 Theoretical interpretation of findings

The relationship between food insecurity and chronic illness observed in this study is best understood through the lens of Syndemic Theory, which conceptualizes the clustering and mutual reinforcement of disease and social disadvantage in structurally vulnerable populations (42). Rather than existing as isolated challenges, food insecurity and chronic disease co-occur in a dynamic interaction shaped by broader structural forces—such as poverty, marginalization, and limited access to care. This framework is particularly applicable to Romani communities in Jordan, who experience a unique intersection of ethnic discrimination, legal invisibility, informal labor conditions, and exclusion from health and social services. Chronic illness reduces individuals’ ability to work and increases out-of-pocket health expenditures, further deepening food insecurity. Conversely, ongoing food deprivation can lead to poor nutrition, compromised immunity, and disease progression, reinforcing a syndemic loop.

Additionally, the Social Determinants of Health (SDH) framework emphasizes the role of upstream societal and policy-level factors—such as geography, marital status, and education—in shaping individual health and nutritional outcomes (43). For instance, the heightened food insecurity observed among participants living in Al-Mafraq or those who were widowed/divorced reflects structural barriers beyond personal choice. Integrating these frameworks provides a more explanatory understanding of how food insecurity among chronically ill Romani adults is not only a reflection of individual health status but also of the social systems in which they are embedded.

### 4.9 Interpreting non-significant associations: cultural and methodological considerations

While prior research has established links between variables such as health insurance coverage and household size with food insecurity risk, our analysis found no statistically significant associations between these factors and food insecurity status among Romani adults with chronic illness. This divergence warrants careful contextual and methodological interpretation.

One possible explanation lies in the cultural and communal dynamics of the Romani population. Unlike nuclear family models often assumed in food insecurity research, Romani households frequently exhibit extended kinship structures characterized by shared caregiving responsibilities, pooled informal income, and collective resource management (27, 44). In such contexts, larger household size may not correspond to increased economic burden. Instead, it may provide resilience against food insecurity through mutual support and resource-sharing practices, such as communal cooking and joint financial decision-making. These patterns have been observed in Roma communities across Eastern Europe and the Middle East, where intra-family networks mitigate the effects of economic hardship in the absence of formal social protections (27).

Similarly, the lack of association between health insurance and food security could reflect discrepancies between coverage and functional access. While approximately half the participants reported having some form of insurance, informal probing during data collection revealed that many were either unaware of how to use their benefits or enrolled in schemes offering only minimal coverage (e.g., emergency services only). These findings suggest that possession of health insurance in marginalized populations does not necessarily equate to improved access or financial protection, thereby limiting its expected buffering effect on food security. Similar mismatches between perceived and actual insurance utility have been reported in other disadvantaged and under-resourced communities (10, 45).

From a methodological standpoint, it is also important to consider the relative homogeneity of our sample. All participants were Romani adults with chronic illness, a subgroup already characterized by overlapping vulnerabilities. This may have minimized between-group variability and obscured associations that could be more detectable in broader or more diverse samples (a phenomenon well documented in prior studies focused on narrowly defined vulnerable populations) (27). Moreover, while our sample was adequate for estimating prevalence, it may have lacked sufficient statistical power to detect small to moderate effect sizes in regression models—particularly for variables with complex or context-dependent influence.

Together, these findings emphasize the importance of culturally informed analysis and the risks of overgeneralizing from models developed in different sociocultural or economic contexts. Future research should aim to integrate ethnographic insights, functional measures of social protection, and culturally adapted indicators to more accurately capture food insecurity risks in Romani and other marginalized populations.

### 4.10 Strengths and limitations

This study offers valuable insights into an under-researched population using validated tools and rigorous statistical methods. The involvement of community leaders and interpreters enhanced participant engagement and data validity. However, several limitations must be acknowledged. The use of a convenience sample limits generalizability beyond the study sites. Self-reported data may be subject to recall and social desirability bias. Furthermore, the cross-sectional design precludes causal inference. Future research should consider longitudinal or mixed-method approaches to explore temporal relationships and lived experiences of food insecurity. Moreover, limitations related to economic measurement necessitate future research that incorporate validated, culturally sensitive tools to assess economic status and livelihood insecurity, particularly in populations with informal or undocumented income sources.

Finally, although covering four governorates enhances geographic representation, the large differences between these regions may introduce variation that is difficult to isolate in the analysis, especially in the absence of a cluster design or representative sampling, therefore we recommend that future research employ stratified or multistage sampling to address this issue more systematically.

### 4.11 Future research

Further studies are warranted to investigate the long-term health and nutritional consequences of food insecurity among Romani and other marginalized communities. Qualitative research can provide deeper insights into cultural norms, coping strategies, and barriers to food access. Comparative studies involving other ethnic minorities or refugee populations in the region could broaden the understanding of intersectional vulnerabilities. Evaluations of pilot interventions—such as integrated health-nutrition clinics or mobile aid services—would also be valuable in guiding policy.

## 5 Conclusion

This study reveals a strikingly high prevalence of food insecurity among Romani adults with chronic illness in Jordan, with more than 90% experiencing low or very low food security. These findings highlight an urgent public health issue within a historically underserved and largely invisible ethnic minority. Food insecurity in this context is deeply interwoven with structural determinants such as poverty, chronic disease, geographic marginalization, and weak institutional inclusion.

To address these challenges, we propose several actionable recommendations. First, the Ministry of Health and the Ministry of Social Development should collaborate to strengthen inclusive health and nutrition programming by designing and implementing mobile outreach services specifically targeting food-insecure Romani households, especially those managing chronic diseases. Mobile clinics and home visits can help overcome barriers related to transportation and lack of official documentation. Second, visibility of Romani populations within national health systems must be enhanced. Governorate-level health directorates and local municipalities should initiate community mapping efforts to document the health and socioeconomic conditions of Romani communities, ensuring that these data are integrated into national registries to inform equitable resource allocation. Third, civil society engagement should be actively supported. Non-governmental organizations (NGOs) and community-based organizations should be mobilized to provide culturally tailored health literacy programs, highlighting the critical connection between nutritional well-being and chronic disease management. Finally, we recommend establishing a multisectoral task force composed of public health officials, researchers, NGOs, and Romani community leaders. This task force would serve to monitor, evaluate, and coordinate comprehensive health equity strategies that effectively address the needs of marginalized populations.

Addressing food insecurity among the Romani population requires more than isolated interventions—it calls for integrated, equity-oriented policies that recognize the structural nature of health and nutritional disparities. Without coordinated action, this population will remain at the periphery of public health planning, with consequences that extend across generations.

## Data Availability

The raw data supporting the conclusions of this article will be made available by the authors, without undue reservation.
